# Bicornuate uterus and pregnancy: ambiguity diagnosis (a case report)

**DOI:** 10.11604/pamj.2022.43.203.32905

**Published:** 2022-12-23

**Authors:** Hamidou Soumana Diaouga, Houegbelo Lazare Laurent, Maimouna Chaibou Yacouba, Fougou Laouane Ari Mamane, Madeleine Garba Rahamatou, Nafiou Idi, Madi Nayama

**Affiliations:** 1 Obstetrics and Gynecology Service, Tahoua Mother and Child Health Center, Tahoua, Niger,; 2 Obstetrics and Gynecology Service, Maternity Issaka Gazobi in Niamey, Niamey, Niger,; 3 Obstetrics and Gynecology Service, Niger-Turkey Friendship Hospital in Niamey, Niamey, Niger,; 4 Obstetrics and Gynecology Service, Maternity of the Regional Hospital of Niamey, Niamey, Niger

**Keywords:** Bicornuate uterus, uterine malformation, pregnancy, case report

## Abstract

The frequency of uterine malformations is estimated in the general population to be between 1 and 4%. The bicornuate uterus accounts for about half of uterine abnormalities. The conception of a pregnancy and its evolution to term on this uterine abnormality is rare. During pregnancy, this malformation is asymptomatic and may go unnoticed in the absence of prenatal care. Ultrasound is essential for the diagnosis, which is often difficult in an environment of socio-economic precariousness and low technical facilities. We present the diagnostic difficulties, the therapeutic aspects and the obstetric prognosis of an unusual case of a unicervical bicornuate uterus revealed during a laparotomy which is both exploratory and diagnostic. Treatment consisted of close monitoring until 38 weeks when the patient underwent a scheduled cesarean section.

## Introduction

The bicornuate uterus is a congenital malformation defect resulting from a defect or failure in the development of the female reproductive system during embryogenesis [1]. The association of pregnancy and uterine malformation is relatively rare. The clinical manifestations are nonspecific, which makes diagnosis difficult in countries with a weak technical platform. Many of them remain asymptomatic and the diagnosis is made only incidentally during an examination performed for another purpose. Thus, it is not exceptional to discover a bicornuate uterus during a first pregnancy check-up or during a vaginal or cesarean delivery [2]. Ultrasound is essential for early diagnosis. Uterine malformation causes pregnancy; high risk pregnancy. Management consists of close monitoring and carrying out pulmonary maturation. Regarding childbirth; cesarean section is the preferred method of delivery [2]. We report the diagnostic difficulties, the therapeutic aspects and the obstetrical prognosis of an unusual case of pregnancy in a bicornuate uterus highlighted during an exploratory laparotomy at the mother and child health center of Tahoua in the republic of Niger.

## Patient and observation

**Patient information:** Mrs. N.M, 31 years old, 4^th^ gesture, 4 parities, mother of two living children. A twin pregnancy was noted in this history during her third full-term pregnancy. She had no history of abortion or premature delivery. She consulted in our department for abdomino-pelvic pain in the context of an unsuccessful pregnancy (5-month amenorrhea).

**Clinical findings:** on examination, there was constipation and intermittent abdomino-pelvic pain. General examination found good hemodynamic and ventilatory status. The obstetric examination found a gravid uterus with a uterine height of 20 cm. Fetal heart sounds (fHS) at 145 beats/min. On vaginal examination, the cervix was short anteriorly dehiscent and fixed under the pubic symphysis. The uterus was globular and painful on mobilization.

**Figure 1:**
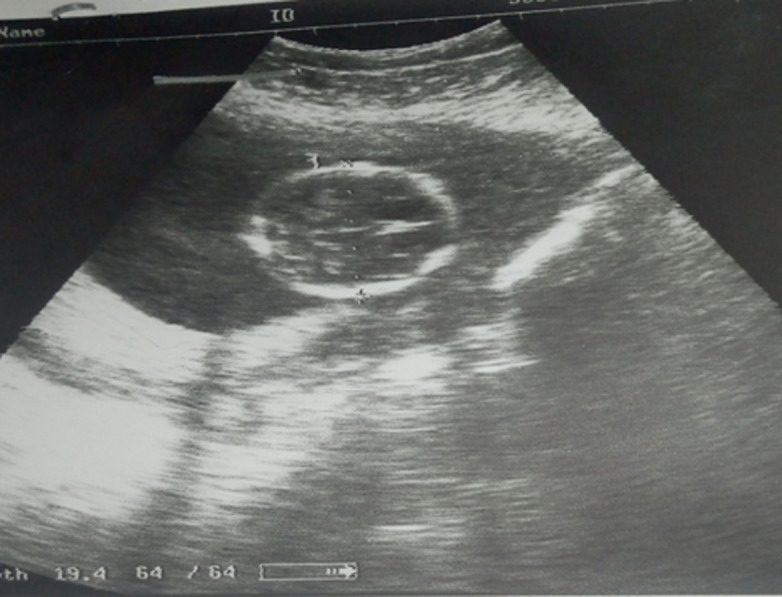
emergency ultrasound; evocative image of a fetus in the abdominal cavity next to an empty uterus

**Figure 2:**
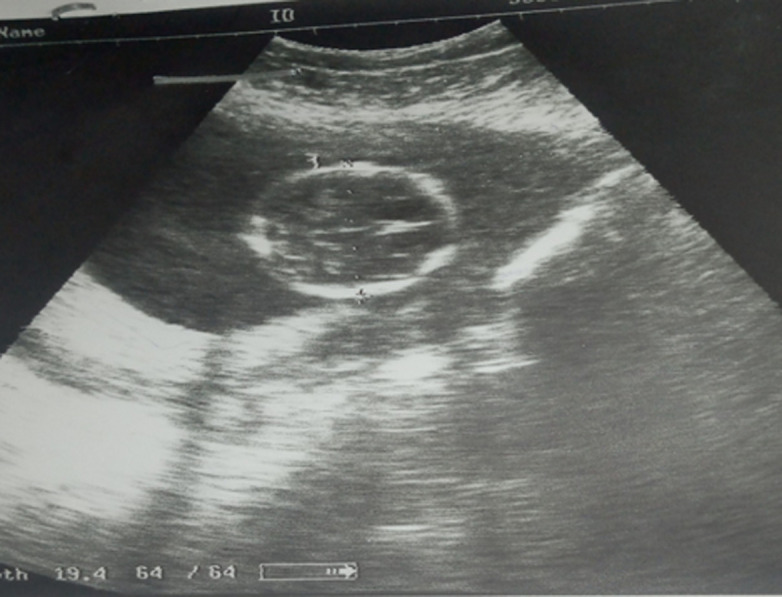
second ultrasound performed: gestational sac with thick wall without fetal anomaly or appendages; next to a non-gravid but globular uterus

**Diagnostic assessment:** the emergency obstetric ultrasound revealed an active pregnancy in the abdominal cavity next to an empty but globular uterus. There was no fetal abnormality or adnexa. The fetal biometry was 20 weeks' amenorrhea (Figure 1). A second ultrasound was requested and noted the same findings as the previous one, however, the wall of the gestational sac was thick (Figure 2). The biological assessment was normal. The differential diagnosis between an abdominal pregnancy and a uterine malformation associated with pregnancy was mentioned. An exploitative laparotomy was then indicated.

**Figure 3:**
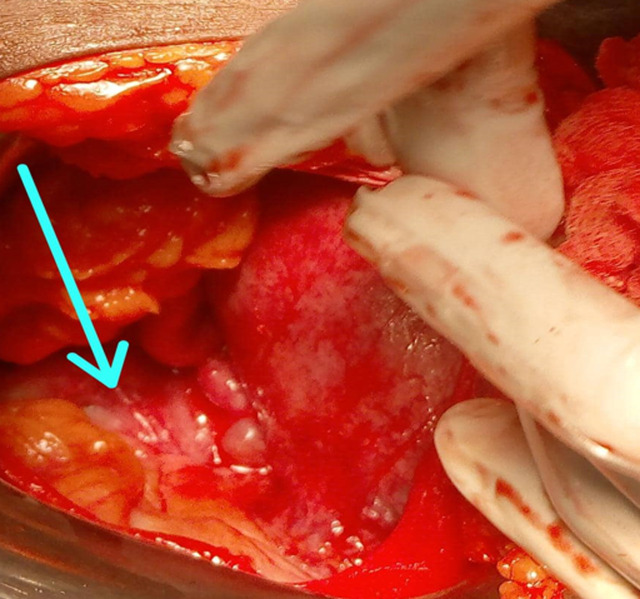
intraoperative discovery of two very distinct completely separate hemi-uteri; the left hemi-uterus is pregnant; the non-pregnant right hemi-uterus is enlarged and globular (arrow); both hemi-uteri have the same cervix

**Diagnosis:** the laparotomy revealed two very distinct separate hemi-uteri, each comprising appendages on only one side, as well as two isthmuses; the first on the left is gravid; the second, on the right, is non-gravid, increased in size and globular. Both hemi-uteri have the same cervix (Figure 3). The abdominal wall was closed plan by plan.

**Therapeutic interventions:** a treatment consisting of injectable phloroglucinol (80mg/8h), natural progesterone (400ug/24h), injectable ceftriaxone (2g/24h in IVD) and metronidazole in infusion (500mg/8h/24h in IVDL) before laparotomy and five days after was administered to the patient.

**Figure 4:**
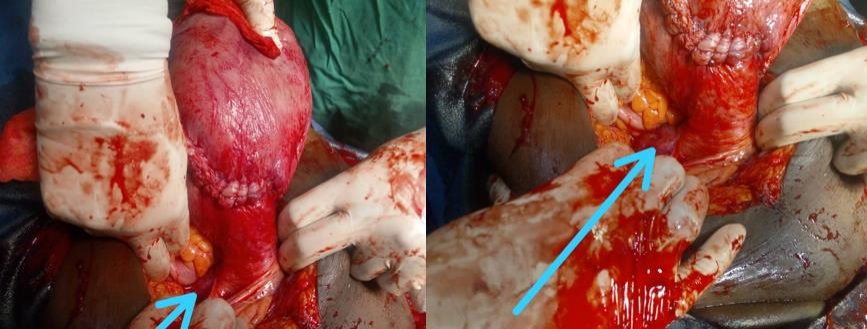
cesarean section scheduled for 38 weeks; image after fetal extraction; unicervical bicornuate uterus; the non-pregnant right hemi-uterus is small (arrow)

**Follow-up and outcome of interventions:** the operative consequences were favorable with discharge on the fifth day. The patient was then seen every two weeks until 38 weeks when she had a scheduled caesarean section (Figure 4). A living child, Apgar 8 and 10, was taken out at the fifth and tenth minute respectively; weight: 2990 grams. The evolution was favorable for the mother and the newborn with exit the 5^th^ postoperative day. Long-distance postpartum radiological ultrasound exploration for associated urinary tract malformations was normal.

**Informed consent:** the patient provided her full consent for publishing her case.

## Discussion

The bicornuate uterus is a uterine malformation associated with the cessation of organogenesis between 10 and 12 weeks of pregnancy, with an abnormal fusion of the two Müllerian ducts [3]. The prevalence of congenital uterine anomalies in the population is estimated between 1 and 4% depending on the studies [4]. Pregnancies carried to term in a malformed uterus are relatively rare [2]. On the embryological level, the organogenesis of the genitourinary tract makes it possible to distinguish four phases: the first, urinary phase (3^rd^, 4^th^ and 5^th^ weeks) comprises the formation of Wolff's ducts and their progression towards the cloaca, the development of ureteral buds in the direction of renal blastemas; the second phase, genital and urinary (6^th^, 7^th^, 8^th^ and 9^th^ weeks) involves the completion of the urinary tract by the ascent and rotation of the kidneys. From the 9^th^ week begins the formation of the Müllerian ducts and their progression towards the genital sinus; the third phase (10^th^, 11^th^ and 12^th^ weeks), genital, involves the joining of the two Müllerian canals; the fourth and last phase is that of resorption of the wall adjoining the Müllerian canals (13^th^ to 17^th^ weeks). The type of malformations is linked to the date of onset of the teratogenic agent during organogenesis: thus between ten and thirteen weeks, the two Müllerian ducts approach the midline. The anomalies observed are a fusion defect of the two Müllerian ducts, at the origin of the two-horned uteri [4]. Several classifications exist for uterine malformations. That of the American Fertility Society (AFS) of 1988 is the most used in the literature [5]. The unicervical bicornuate uterus as in our observation corresponds to class IV. These uterine malformations are often accompanied by urinary malformations. In our patient, the remote assessment did not reveal any urinary tract abnormalities.

Clinically, bicornuate unicervical uteri remain asymptomatic; the diagnosis is only made incidentally during an examination performed for another purpose or during a vaginal delivery in front of obstructed labor or during a cesarean section [5]. Paraclinically; during pregnancy ultrasound is essential for diagnosis. However, when the pregnancy is advanced, ultrasound rarely provides the diagnosis [6]. Indeed, the two obstetric ultrasounds performed on our patient were inconclusive. Nuclear Magnetic Resonance Imaging (MRI) is the most powerful test; however not accessible in our context. A group of signs found in our patient suggests an abdominal pregnancy in the foreground [7], namely: digestive disorders: constipation; abdomino-pelvic pain concomitant with uterine mobilization; palpation of a second pelvic mass corresponding to the uterus enlarged but empty; vaginal examination, the cervix fixed under the pubic symphysis, our patient had also carried out three previous pregnancies to term including a twin pregnancy all delivered vaginally without any notion of obstructed labor.

This situation is exceptional in uterine malformations. However, we found other signs in favor of the uterine malformation: the globular uterus with a short cervix and dehiscent on vaginal examination; and on ultrasound: absence of fetal anomaly and adnexa, normal amount of amniotic fluid, thickness of the gestational sac near a non-gravid but globular uterus. This diagnostic ambiguity led us to perform an exploratory laparotomy. From an obstetrical point of view, congenital uterine malformations represent 5-10% of the causes of repeated miscarriages and 25% of late miscarriages or premature deliveries [2]. The problem in patients with these malformations is not that of conceiving, but of carrying the pregnancy to term. Several factors explain this: a reduced uterine cavity, less efficient uterine musculature, an inability to distend the uterus, myometrial and cervical dysfunction, inadequate vascularity and a poorly developed endometrium [2]. Overall, uterine malformations are responsible for a large number of obstructed presentations (23-61% of breech presentations) [3].

In the case of a bicornuate uterus, the fetus may position the wider part of its body towards the maternal pelvic region, giving it more space. This is why the presentation rate “in siege” is higher [2]. Regarding management, When the diagnosis of uterine malformation is made at the start of pregnancy, treatment will only be preventive (rest, lung maturation, ultrasound monitoring of fetal growth and cervical competence) [2,8]. The role of cervical cerclage is not certain in the prevention of preterm delivery, except in cases of incompetence of the cervix documented by ultrasound or hysterosalpingography and the history of previous preterm deliveries which was not the case in our patient. In fact, cases of pregnancies at term or near term without cervical cerclage with a unicorn or bicornuate uterus have been reported in the literature [2,9,10]. Our observation corroborates these data. In the term; the high frequency of obstructed labor makes caesarean section the preferred route of delivery [2,3,9,10]. Indeed, in our patient; despite the previous history of vaginal eutocic delivery, we preferred to perform a cesarean section.

## Conclusion

A bicornuate uterus does not always lead to obstetric complications. He can carry pregnancies to term including twin pregnancies. Diagnosis is not always easy, especially in our under-equipped countries. It is important to make an ultrasound diagnosis at the start of pregnancy in order to manage the situation preventively and to allow the extraction of the fetuses in good conditions before any complications. In case of uterine malformation; cesarean section is the preferred method of delivery.
